# The glycan fingerprint in immune thrombocytopenia

**DOI:** 10.1016/j.rpth.2026.103388

**Published:** 2026-02-16

**Authors:** Vivianne S. Nelson, Jan Zlamal, Rick Kapur

**Affiliations:** 1 Sanquin Blood Supply Foundation, Department Research, and Amsterdam UMC location University of Amsterdam, Landsteiner Laboratory, Amsterdam, The Netherlands; 2 Department of Hematology, Haga Teaching Hospital, the Hague, The Netherlands; 3 Department of Hematology, Leiden University Medical Center (LUMC), Leiden, The Netherlands; 4 Institute for Clinical and Experimental Transfusion Medicine, University Hospital of Tübingen, Tübingen, Germany; 5 Centre for Clinical Transfusion Medicine, University Hospital Tübingen, Tübingen, Germany

**Keywords:** Fc-glycosylation, glycobiology, glycosylation, immune thrombocytopenia, ITP, platelet autoantibodies, platelet desialylation

## Abstract

A state-of-the-art lecture titled “Platelet Glycobiology and Immune Thrombocytopenia (ITP)” was presented at the International Society on Thrombosis and Haemostasis (ISTH) congress in 2025. In this review, we summarize the current knowledge on glycan-mediated mechanisms in ITP, including glycan alterations of platelet autoantibodies and on the surface of platelets and megakaryocytes. We discuss how these glycan modifications might contribute to thrombocytopenia, and potentially to an increased risk of bleeding in patients with ITP. We address relevant new data on this topic and emphasize key directions for future ITP glycobiology research.

## Essentials

• Antiplatelet glycans and glycans on platelets and megakaryocytes may shape ITP pathophysiology.
• IgG Fc-glycosylation composition may affect platelet clearance in a subset of patients with ITP.
• Both anti-GPIb/IX and anti-GPIIb/IIa can induce platelet surface desialylation in ITP.
• Glycan analysis could indicate ITP disease severity but warrants more investigation.

## 1 Introduction

Patients with immune thrombocytopenia (ITP) suffer from low platelet counts and increased bleeding tendency due to autoimmune dysregulation targeting platelets and megakaryocytes [[1], [2], [3], [4]]. The pathophysiology of ITP is heterogeneous and involves a complex interaction of immune tolerance loss, aberrant B and T cell responses and activation of antigen-presenting cells [[1], [2], [3], [4]]. IgG platelet autoantibodies are commonly found in patients with ITP [5,6]. These autoantibodies are directed against glycoprotein (GP) complexes on the platelet surface, most commonly anti-GPIIb/IIIa, GPIb/IX, and GPV [2,4,5]. Binding of the fragment crystallizable (Fc) domain of anti-GP induces Fcγ-receptor (FcγR)-mediated phagocytosis by macrophages in the spleen and/or liver [2,4,5]. Moreover, binding of C1q by anti-GP promotes complement activation causing platelet phagocytosis or cytotoxicity [2,4,5]. Glycans are a critical part of the antibody structure and are added to antibodies, or other proteins, as a posttranslational modification [7,8]. Two types of glycans have been described: N-glycans and O-glycans. N-glycans bind to proteins at asparagine residues and consist of a *N-*acetylglucosamine (GlcNac) and mannose bi-antennary core structure [7,8]. The sugar moieties beyond this core structure differ in composition and can include fucose, *N*-acetylgalactosamine (GalNAc), galactose, and/or sialic acid residues in various degrees [7,8]. O-glycans are attached to proteins at serine or threonine residues and their core structure is often shorter and less branched than that of N-glycans [7,8]. For IgG1, IgG2, and IgG4 antibodies, there is one specific N-glycan site at the Fc-domain [9,10]. The composition of this Fc-glycan is highly variable, which affects the antibody affinity and subsequent effector functions [[9], [10], [11]]. IgG-glycans have been shown to orchestrate antibody responses in various inflammatory and immune diseases [9,12], and may also contribute to the ITP pathophysiology through influencing key antibody-mediated processes resulting in platelet clearance. The anti-platelet Fc-domain, however, is not the only site where glycan composition could be functionally relevant in ITP. The loss of sialic acids from the platelet surface, platelet desialylation, has been described to promote their clearance through the hepatic Ashwell-Morell receptor (AMR) [[13], [14], [15]]. Although desialylation is a physiological process during platelet aging, extensive desialylation, induced by anti-GP or possibly CD8^+^ T cells, has been described to accelerate platelet destruction in ITP [14,[16], [17], [18]]. This state-of-the-art review explores the impact of glycobiology on the ITP pathophysiology, focusing on glycosylation of platelet autoantibodies as well as glycans on the platelet and megakaryocyte membrane. We will reflect on new findings presented during the 2025 International Society on Thrombosis and Haemostasis congress and aim to give directions for future research on antibody and platelet glycobiology in ITP.

## 2 Part 1: Platelet Autoantibody Glycosylation

Total IgG Fc-glycosylation profiles have been studied in ITP. A study investigating the total IgG of responding and non-responding patients to splenectomy, found that the IgG1 Fc-fucosylation was reduced in patients with ITP. However, following splenectomy, the IgG1 Fc-fucosylation levels rose to match those seen in healthy individuals [19]. Comparable levels of total IgG1/2/3 Fc-galactosylation and sialylation were observed between patients with ITP and healthy controls, although there was a high variability among all groups [19]. Prior work showed similar total IgG Fc-glycosylation profiles in patients with ITP and a relapse after corticosteroids therapy compared to healthy controls [20]. Furthermore, B cell depletion through anti-CD20, rituximab, only slightly altered the total IgG1 Fc-glycosylation status, and these alterations did not correlate to the platelet response to rituximab [20]. While these total IgG studies may offer some insights, their interpretability and generalizability remain limited, as IgG Fc-glycosylation is strongly antigen- and context-dependent. There is substantial interindividual variability in Fc-glycosylation patterns, with age, genetics, hormonal changes and lifestyle factors playing major roles [9]. Additionally, most of the circulating IgGs lack antigen engagement and their predominance in total IgG profiling might mask disease- and antigen-specific IgG responses [21]. Moreover, IgG glycosylation is separately regulated for each antigen and may dynamically change over time depending on the disease context [21]. Therefore, to actually understand the dynamics of IgG Fc-glycosylation in ITP, platelet-specific IgG Fc-glycosylation analysis should be performed. On the other hand, the total IgG glycosylation status might still be of importance, as it is hypothesized to modulate the binding and function of specific pathogenic antibodies [10]. Hence, it is essential to look at antigen-specific IgG Fc-glycosylation in the context of the total IgG Fc-glycosylation status, disease characteristics, and other factors known to affect glycosylation. While age and sex was matched in the total IgG Fc-glycosylation studies in ITP, no correction was performed for platelet counts. No antigen-specific IgG analysis has, to the best of our knowledge, yet been performed in ITP. This is likely related to challenges in adequately purifying antigen-specific IgG in ITP. These challenges arise partly from the relatively low levels of platelet autoantibodies (compared to platelet alloantibodies) and perhaps partly from the platelet-FcγRIIA receptor that can bind IgG non-specifically. Hence, newer techniques for purification of platelet autoantibodies are therefore warranted to perform robust platelet-antigen-specific Fc-glycosylation analysis in ITP.

Contrary to ITP, platelet-antigen-specific IgG Fc-glycosylation has been studied in fetal and neonatal alloimmune thrombocytopenia (FNAIT). FNAIT is characterized by the maternal production of platelet alloantibodies, most commonly anti-human platelet antigen-1a (anti-HPA-1a), that cross the placenta and target the platelets of the fetus and newborn [22,23]. Strikingly, a decrease in anti-HPA-1a Fc-fucosylation was observed in 48 FNAIT cases, compared to total IgG [23], which was subsequently confirmed in 166 patients with FNAIT [22]. Mechanistically, afucosylated anti-HPA-1a was shown to have a stronger binding affinity to FcyRIIIA and FcγRIIIB which consequently enhanced platelet phagocytosis *in vitro* [23]. Clinically, a lower anti-HPA-1a Fc-fucosylation also correlated with lower neonatal platelet counts and with clinical disease severity, however, not for all cases [23]. Anti-HPA-1a Fc-galactosylation was also found to be increased in FNAIT [22,23]. In one study, increased anti-HPA-1a Fc-galactosylation together with decreased anti-HPA-1a Fc-fucosylation and increased levels of anti-HPA-1a, correlated with lower platelet counts and a higher bleeding risk [22]. General *in vitro* studies to IgG glycosylation have shown that increased IgG Fc-galactosylation leads to more IgG hexamerization [24]. This hexamerization of IgG leads to a greater capacity to bind C1q and subsequent activation of the classical complement pathway [24,25]. This mechanism was confirmed for 2 platelet alloantibodies (anti-HLA class I and anti-HPA-1a), in which increased Fc-galactosylation resulted in increased complement activation on platelets *in vitro* [26]. Contrary to Fc-galactosylation, increased IgG1 Fc-sialylation was shown to prevent C1q binding to the Fc-domain and thereby impair complement-mediated cytotoxicity, independently from antigen specificity [27]. Further investigation of anti-HLA in an alloimmunized cohort after platelet transfusions revealed highly variable Fc-glycosylation profiles [28]. Higher galactosylation and sialylation of anti-HLA class I correlated with lower platelet count increments after transfusion in this cohort, while no correlation to the number of administered platelet transfusions was found [28].

Taken these alloimmune studies together, increased Fc-galactosylation along with decreased Fc-fucosylation may reflect a glycosylation signature linked to higher platelet phagocytosis *in vitro* and lower platelet counts *in vivo*. Given the pathophysiological similarities between FNAIT and ITP, and the findings of the total IgG glycosylation studies in ITP, antigen-specific Fc-glycan analysis may also yield clinically relevant outcomes in ITP. The pathophysiological relevance of the IgG Fc-glycan in ITP has in fact already been demonstrated, as complete IgG deglycosylation inhibited antibody-induced platelet phagocytosis *in vitro* and *in vivo* in a passive ITP mouse model [29]. Moreover, a notable attempt has been made to hypersialylate intravenous immunoglobulins (IVIG) for patients with ITP with acute bleeding symptoms. Some studies have proposed that Fc-sialylation is essential for IVIG’s anti-inflammatory effect by binding to C-type lectins (eg, the DC-SIGN receptor or CD23) [8,30,31]. However, contradictory evidence exists, with other studies showing no IgG interaction with DC-SIGN or CD23, indicating that the mechanism of IVIG remains to be elucidated [32]. A phase 1/2 clinical trial compared a hypersialylated IgG product derived from IVIG, M254, with “normal” IVIG treatment in patients with ITP [33]. Despite preclinical data showing a nearly 10-fold higher activity of M254, this trial revealed that efficacy in terms of platelet response was lower for M254 compared to IVIG and the study was terminated prematurely [33] (clinicaltrials.gov: NCT03866577).

## 3 Part 2: Platelet Membrane Glycosylation

### 3.1 Physiological platelet glycan-driven senescent platelet clearance

**Figure 1:**
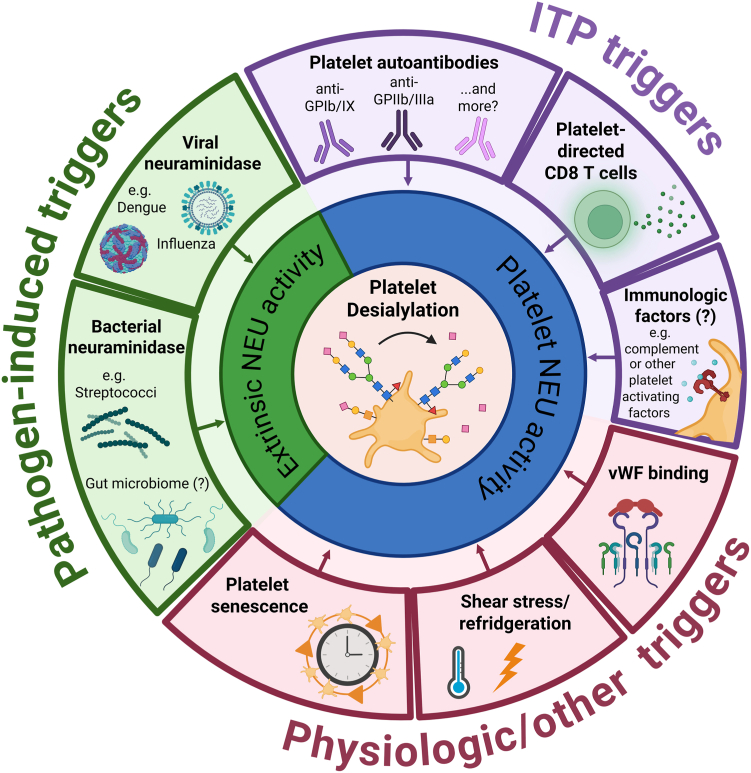
Triggers for platelet desialylation. The question mark (?) indicates that more research is required to validate this hypothesis. GP, glycoprotein; ITP, immune thrombocytopenia; NEU, neuraminidase; VWF, von Willebrand factor. Created in BioRender. Kapur, R. (2026) https://BioRender.com/p20imc3.

Platelets express various surface GPs that are essential for platelet function [34]. GPIb stands out as the most heavily glycosylated protein and carries up to 48 different O-glycosites and 1 N-glycosite, predominantly capped with sialic acid [15,35]. Sialic acids play a pivotal role in dictating platelet lifespan and regulating platelet function [13,36,37]. Aging platelets lose their sialic acid, which is recognized by the hepatic AMR or the macrophage C-type lectin receptors, resulting in platelet clearance [13,[38], [39], [40], [41]]. The binding of desialylated platelets to the AMR triggers a JAK2-STAT2 signaling pathway, which has been shown to result in hepatic synthesis of thrombopoietin (TPO) *in vitro* and *in vivo* [13,42]. This is a potential feedback mechanism in which the clearance of senescent platelets boosts TPO levels and stimulates thrombopoiesis by megakaryocytes in the bone marrow niche. Follow-up research indicated that the interaction between platelet delta-like 4 and the Notch1 receptor, which is in proximity to the AMR, on hepatocytes is also involved [43]. Kupffer cells, hepatic resident macrophages, may also contribute to TPO generation by facilitating platelet crossing through the hepatic endothelial barrier [44]. Alternatively, Kupffer cells are also capable of directly phagocytosing desialylated platelets by binding their αMβ2 integrins (CD11b), macrophage galactose lectin, and/or CLEC4F receptor to platelet-GlcNac or galactose residues [38,39,45,46]. The exact mechanisms through which senescent platelets interact with hepatic cells remain to be elucidated [47]. The liver is likely not acting alone in senescent platelet clearance, as splenic macrophages also express macrophage galactose lectin and CD11b and therefore are able to recognize desialylated platelets [45]. This is illustrated in a recent study focusing on platelet sequestration patterns in healthy individuals, where 50% (5/10) had a splenic sequestration pattern [48]. A recent animal study, however, found that clearance of desialylated platelets in mice was almost exclusively performed in the liver, predominantly by macrophages [49]. Moreover, knock-out of platelet-GPIb-α and the AMR in this study resulted in an increase of desialylated platelet pooling in the spleen, but had no effects on other platelet kinetics [49]. Platelet desialylation is predominantly mediated by the translocation of neuraminidases (NEU), enzymes that cleave sialic acid, to the platelet surface [36]. Four types of neuraminidase (NEU1-4) have been identified in platelets [50,51]. In resting platelets, NEU2 is located in α-granules while NEU1 co-localizes with mitochondria [36]. Upon platelet activation, eg, after rewarming refrigerated platelets or after binding of von Willebrand factor (VWF) to GPIb-α, these NEUs are translocated to the platelet membrane [36,52]. Multiple other triggers for increased NEU expression on the platelet surface have been identified or hypothesized, both in physiological and pathological settings (Figure 1). Apart from endogenous NEU translocation, bacterial or viral NEUs also induce platelet desialylation, promoting thrombocytopenia in infected individuals [[53], [54], [55], [56]] (Figure 1).

### 3.2 Platelet desialylation in ITP

#### 3.2.1 Triggers of platelet desialylation

Nieswandt et al. [57] initially proposed a Fc-independent platelet depletion mechanism in murine ITP, with the passive murine ITP model of Li et al. [15], based on the injection of anti-CD41, revealing the relevance of platelet desialylation. In that study, monoclonal anti-GPIb-α not only induced P-selectin expression, but also enhanced NEU1 translocation and subsequent platelet desialylation both *in vitro* and *in vivo* [15]. Additionally, they demonstrated that anti-GPIbα-mediated platelet clearance mainly occurred in the liver by the AMR [15]. Interestingly, while they observed a high desialylating potential of murine and human anti-GPIb, no anti-GPIIb/IIIa-mediated platelet desialylation was detected [15]. Several follow-up studies, however, have indicated that ITP-mediated desialylation is not restricted to antibodies targeting GPIbα [17,58,59]. In these studies, anti-GPIIb/IIIa harboring ITP patient sera also induced platelet desialylation, potentially via the engagement of FcγRIIA on the human platelet surface [17,58]. As murine platelets lack the platelet FcγRIIA receptor, this could explain why, in the models of Li et al. [15], no effect of monoclonal GPIIb/IIIa was observed, while in the humanized mice model of Marini et al. [17], both anti-GPIb/IX and anti-GPIIb/IIIa led to increased platelet clearance. Interestingly, only a subset (approximately one third) of anti-GPIb/IX and anti-GPIIb/IIIa was detected for having the capacity to desialylate platelets *in vitro* [17,58,59]. Zheng et al. [58] tested 2 sera containing anti-GPV and found no desialylation. Furthermore, platelet desialylation was also induced by ITP sera without detectable platelet autoantibodies [17,59]. While those findings could be explained by the presence of ITP antibodies that remain undetected by current antibody detection assays, other factors in ITP sera might also induce platelet desialylation. ITP patient-derived cytotoxic CD8^+^ T cells have also been described cause platelet desialylation and platelet uptake by hepatocytes *in vitro* [18]. Furthermore, infusion of CD8^+^ splenocytes in mice in this study also led to increased platelet desialylation and clearance in the liver [18]. As no subsequent studies have been performed, the underlying mechanisms remain unclear, although CD8^+^ T cells have been shown to express NEU1 and NEU3 [60]. Additionally, as platelet activation is likely required for NEU translocation, other platelet-activating triggers might also be involved. While ITP data about the association between platelet activation and desialylation is inconsistent [[61], [62], [63], [64]], future studies should elucidate whether other triggers besides antibodies and CD8^+^ T cells play a relevant role in platelet desialylation in ITP, Figure 1.

#### 3.2.2 Intracellular signaling pathways of platelet desialylation

Deeper knowledge regarding the underlying mechano-molecular signaling pathways that contribute to platelet desialylation in ITP is lacking. A study performed by Quach et al. [65] suggests that anti-GPIbα-mediated platelet desialylation might be caused via antibody-mediated unfolding of the subunits’ lectin-binding domain, which mediates calcium-dependent platelet activation similar to that observed following VWF binding [66]. The cytoplasmic domain of GPIb-α is associated with the adaptor/modulator protein 14-3-3ζ, which mediates rapid GPIb-α mechano-molecular signal transduction, resulting in platelet secretion and integrin activation during hemostasis [67,68]. Several signaling molecules, including SRC-family kinase Lyn, Rac-1, or Akt, have been reported to be essential in the GPIb-α-mediated platelet activating signaling pathway [[69], [70], [71]]. In the context of ITP, GPIb-α-antibodies have been reported to induce Akt-mediated platelet apoptosis through activation of phosphodiesterase-mediated inactivation of anti-apoptotic protein kinase A [72,73]. Interestingly, a sialidase inhibitor was shown to prevent anti-GPIbα–induced platelet clearance *in vivo* [72,73]. However, a direct link between activated Akt and platelet desialylation has not been established yet.

Apart from anti-GPIbα-mediated platelet desialylation, a growing body of evidence suggests that a subgroup of anti-GPIIb/IIIa antibodies shows significant potential to cause platelet desialylation via Fc-mediated crosslinking of platelet FcγRIIA [17,58]. While this is a long-standing concept in the pathophysiology of heparin-induced thrombocytopenia and other anti-PF4-related diseases [74,75], its role in ITP remains less clear. In fact, pharmacological targeting of critical mediators in the platelet FcγRIIA-mediated signaling downstream could be a feasible approach to prevent ITP antibody-induced platelet desialylation [17,58]. Targeting platelet spleen tyrosine kinase (Syk) or Bruton’s tyrosine kinase was recently shown to prevent antibody-induced platelet activation in different FcγRIIA-mediated prothrombotic diseases [[76], [77], [78]]. While Syk inhibition is implemented as a safe treatment approach in current ITP treatment guidelines [79], more evidence regarding the safety profile of Bruton’s tyrosine kinase inhibition is required. The exact contribution of the distinct signaling enzymes to platelet desialylation remains not well elucidated yet. Future work should be dedicated to further elucidating the FcγRIIA-mediated pathway in ITP.

#### 3.2.3 Platelet desialylation affecting coagulation

The extent to which platelet desialylation induces functional changes potentially affecting coagulation in ITP remains unclear. On the one hand, platelet desialylation has been associated with an activated and/or apoptotic platelet phenotype, which is usually procoagulant [80,81]. On the other hand, desialylation accelerates platelet clearance, and desialylating antibodies were shown to impair adhesion of fibrinogen and VWF to platelets, contributing to more bleeding [17]. Additionally, a clinical association between having antibodies with desialylating capacity and bleeding symptoms in ITP was suggested in 2 studies [17,59]. However, in the study by Schramm and colleagues, this association was likely confounded as its significance disappeared in multivariate models accounting for differences in platelet counts [59]. Additionally, the antibody detection methods in this study were suboptimal [59]. Overall, further studies are necessary to clarify whether platelet desialylation is associated with a more thrombotic or bleeding phenotype.

#### 3.2.4 Platelet desialylation affecting immune regulation

As sialic acids are also ligands for immune cells, it could be of interest to investigate whether platelet desialylation affects immune response [41]. Siglec-receptors are inhibitory receptors expressed on macrophages and leukocytes that use sialic acid on the cell’s surface to discriminate between self and non-self [82,83]. Thus far, platelet desialylation and the effect on Siglec-receptor engagement have not been studied in ITP. Furthermore, desialylation of O-glycans on platelet GPIIb led to a higher antigenicity of the HPA-9b epitope with stronger binding of anti-HPA-9b [84]. Hence, the authors hypothesize that the epitope might also be less sialylated at the time of immunization. In a murine alloimmunization study, it was described that transfusion of desialylated platelets, both in β3 and GPIβ knock-out mice, led to lower alloantibody titers compared to wild-type platelet transfusion [49]. The study shows that Kupffer-cell-mediated clearance of desialylated platelets might drive this immunosuppressive effect through the induction of anti-inflammatory cytokines [49]. However, reduced antigen exposure due to faster clearance of desialylated platelets may also potentially contribute in this setting. The potential immunogenicity of platelet glycan exposure in ITP has yet to be investigated. Patients with ITP have a dysregulated immune response, which is marked by a decrease in regulatory T cells (Tregs) [[2], [3], [4]]. Interestingly, an inverse correlation between the platelet desialylation rate and the number of Tregs was shown in patients with ITP [16]. This, however, remains an association, and a potential mechanistic link remains to be studied, as both markers could also reflect a more severe disease state, with desialylation being a consequence of the dysregulated immune response in ITP.

#### 3.2.5 Platelet desialylation affecting platelet sequestration site and circulating TPO levels

While the aforementioned mouse model data indicate that desialylated platelets are also cleared via the AMR or macrophages in the liver in the setting of ITP [15], clinical evidence is lacking. Numerous studies have explored the association between platelet sequestration site and antibody specificity, but without specifically looking at platelet desialylation [[85], [86], [87]]. None of these studies have reported an association between anti-GPIb/IX and hepatic sequestration specificity [[85], [86], [87]], except for one sub-analysis of severe thrombocytopenic patients (<50 × 10^9^/L) [88]. This analysis also observed a positive association between anti-GPIb/IX and TPO levels in this ITP cohort with severe thrombocytopenia [88]. At least, these data indicate that anti-GPIb/IX does not seem to block TPO production in patients with ITP. While preclinical physiologic studies have shown a feedback loop between platelet desialylation and hepatic TPO synthesis [13,42], only one study has investigated this correlation in patients with ITP [64]. This study found that baseline TPO levels tended to be elevated in patients with refractory ITP who had higher desialylation levels compared to non-refractory ITP [64]. However, the TPO levels did not correlate to antibody specificity, while within the refractory group, anti-GPIb did correlate to higher desialylation levels [64]. Moreover, another study found an association between the presence of antibodies (both anti-GPIb/IX and anti-GPIIb/IIIa) with desialylation capacity and high TPO levels (>50 pg/mL) in their univariate, but not their multivariate analysis [59]. All in all, the effect of platelet desialylation on platelet sequestration patterns and TPO levels in ITP should be further investigated in a clinical setting.

#### 3.2.6 Platelet desialylation as a diagnostic or prognostic marker

The question is whether platelet desialylation could serve as an additional diagnostic or prognostic marker for ITP. The rate of platelet desialylation can be assessed by either mass spectrometric analysis or flow cytometry using glycan-binding lectins [37,40]. A consensus protocol using 2 fluorescein-labeled lectins (*Ricinus communis* agglutinin-1, RCA-1; and *Erythrina cristagalli* lectin, ECL) to measure galactose-β on the platelet surface was recently published [37]. Some studies demonstrate that, at the group level, platelet desialylation is generally elevated in ITP [89,90]. However, if we look within the ITP cohort, increased platelet desialylation levels are only observed in patients with low platelet counts [62] or patients who are refractory to therapy [16]. Moreover, when ITP is compared with other non–ITP thrombocytopenia-associated diseases, comparable levels of platelet desialylation are found [63,89,90]. Platelet desialylation does, therefore, not appear to be a specific diagnostic marker for ITP. Platelet counts have been correlated with the rate of platelet desialylation in multiple studies [17,62,90], except for one [59]. Accordingly, one could speculate whether desialylation is a marker of (severe) thrombocytopenia in general, rather than being specific for ITP disease flares.

Platelet desialylation has been correlated with therapy refractoriness in ITP, although there is a lot of heterogeneity between studies in how refractory ITP is defined [16, 64, 89, 91]. Furthermore, the major limitation of these studies is that they did not correct for platelet counts. In most studies, the refractory ITP group had lower platelet counts compared to the other included ITP cohorts at the time of measurement [16, 64, 89]. This raises the question whether platelet desialylation is just a marker of (general) thrombocytopenia. One study did not report differences in platelet counts when comparing responders to non-responders [91]. This study specifically looked at patients with a splenic sequestration pattern not responding to splenectomy and found increased RCA-1 expression for this group [91]. They also checked for diverse platelet apoptotic and activation markers like caspase-3,7,8,9, P-selectin and PAC1, but found no differences in these markers between responders and non-responders [91]. To conclude, so far there is not enough solid evidence that platelet desialylation could serve as a diagnostic or prognostic marker in ITP. While some promising findings exists, only a longitudinal assessment of sialic acid with a measurement before and after therapy and with correction for platelet counts could confirm its relevance as a prognostic marker.

#### 3.2.7 Platelet desialylation as a therapeutic target

Oseltamivir is an inhibitory drug that targets influenza neuraminidase. Despite its limited activity against human (platelet) sialidases, there have been several studies showing that the drug might have an additional beneficial effect for patients with ITP [[92], [93], [94]]. One study showed that oseltamivir combined with dexamethasone led to a higher response rate at 6 months compared to dexamethasone alone in newly diagnosed patients (53% vs 30%) [95]. Positivity for anti-GPIb did not predict a response [95]. To date, identifying which patients might benefit from oseltamivir remains difficult due to the absence of predictive markers and the lack of efficacy data for combinations with other treatments. Besides oseltamivir, other ways to inhibit platelet NEU have been investigated. A study investigating Staphylococcus aureus bacteremia found that ticagrelor, a commonly prescribed P2Y12 platelet receptor inhibitor, was also able to inhibit platelet sialidase activity [55]. However, in the setting of ITP, inhibition of platelet activation may not be desirable, as it could increase the risk of bleeding.

## 4 Part 3: Megakaryocyte Membrane Glycosylation

Knock-out mice missing critical enzymes for O- and N-glycan formation have profound thrombocytopenia and impaired proplatelet formation, underscoring the importance of glycan structures on megakaryocytes [41,96,97]. Desialylating antibodies in ITP sera were shown to inhibit adhesion of megakaryocytes to the extracellular matrix of the bone marrow, ultimately resulting in impaired proplatelet formation [17]. In this study, 80% (8/10) of the ITP sera were able to desialylate megakaryocytes, which had both anti-GPIb and anti-GPIIb/IIIa specificity [17]. In another study, only 1 (out of 20, 5%) ITP serum, which contained anti-GPIb, was able to induce megakaryocyte desialylation, while inhibition of thrombopoiesis was more often observed (13/20, 65%) [61]. One study hypothesized that Siglec-receptors on bone marrow immune cells engage in recognizing sialic acid loss on O-glycans on megakaryocytes, leading to the subsequent secretion of IFN-γ and inhibition of platelet production [98]. Given the preliminary character and small numbers of these studies, the role of megakaryocyte glycan desialylation in the impaired thrombopoiesis in ITP should be further investigated.

## 5 ISTH Congress Report

The state-of-the-art session on ITP during the 2025 ISTH congress (SOA 04 – Immune thrombocytopenia) featured 3 talks, which were all highly complementary. The first talk (by Dr Annemarie Fogerty) elaborated on the methods and our current understanding of platelet autoantibody testing in ITP. The second talk (by Dr Rick Kapur, and subject of the current article) focused on the role of glycobiology in the pathophysiology of ITP, discussing both glycans on platelet autoantibodies, as well as glycans on the surface of platelets and megakaryocytes. The third talk (by Dr Thomas Pincez) discussed the immunopathology of ITP from a broader perspective, focusing on both platelet autoantibody and cellular responses.

## 6 Conclusion and Future Directions

**Figure 2:**
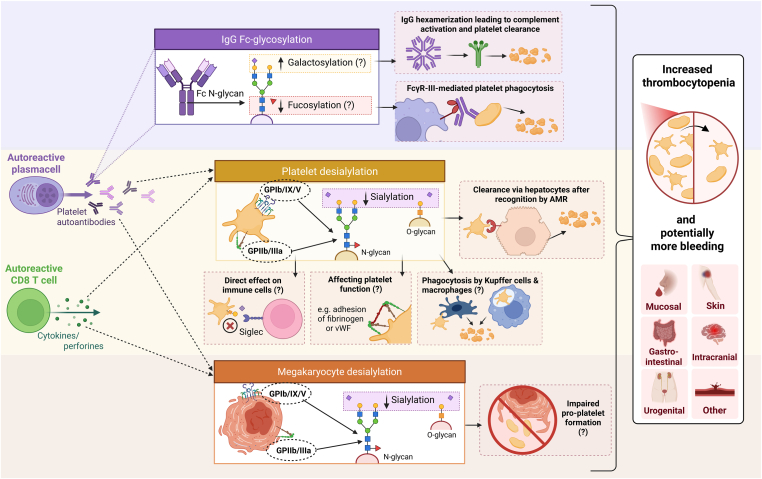
The glycan fingerprint in the pathophysiology of ITP. The question mark (?) indicates that more research is required to validate this hypothesis. Fc, fragment crystallizable; FcγR, Fc-gamma-receptor; GP, glycoprotein; IgG, immunoglobulin G; ITP, immune thrombocytopenia. Created in BioRender. Kapur, R. (2026) https://BioRender.com/4n5kv5w.

The ITP glycan fingerprints, as discussed in this review, along with their potential functional consequences, are summarized in Figure 2. Taken together, evidence from studies on total IgG Fc-glycosylation in ITP and on alloimmune platelet-antibodies both suggest that, at least in a subset of patients, decreased Fc-fucosylation and increased Fc-galactosylation might indicate a more severe disease state. However, research into platelet autoantibody-specific IgG Fc-glycosylation in ITP is lacking and warranted. Furthermore, future studies should account for important contextual factors like disease phase (platelet counts), treatment, age, sex, and lifestyle-related factors. Beyond Fc-glycosylation, roughly 10% to 20% of IgG antibodies also carry N-glycans within the variable region of their fragment antigen-binding (Fab) domain [9]. It has been proposed that autoreactive B cells in some diseases selectively insert Fab-glycans during somatic hypermutation, which results in Fab-glycan enrichment [9,99]. The specific role of IgG Fab-glycosylation in ITP remains to be determined. In addition to the IgG Fc-glycosylation studies already discussed, 2 studies investigated total IgG N-glycan profiles in ITP (*n* = 34 and *n* = 56) versus healthy controls (*n* = 35 and *n* = 26) [100,101]. Both studies reported a decrease in total IgG N-glycan galactosylation for patients with ITP [100,101]. Interpretation of these results is challenging, as the impact of Fab glycosylation on these profiles remains uncertain. To substantially advance antibody glycosylation research in ITP, future efforts should focus on developing methods to isolate platelet autoantibodies without the interference of aspecific antibodies bound to the platelet FcγRIIA receptor.

Both anti-GPIb/IX and anti-GPIIb/IIa have been shown to induce platelet desialylation in ITP, yet interestingly, only a subset of these antibodies exhibits desialylating capacity. This heterogeneity should be further researched to shed light on the desialylating potential of platelet autoantibodies beyond their antigen specificity. Future investigations should prioritize elucidating the mechano-molecular signaling pathways triggered by platelet autoantibodies that result in platelet desialylation. Additionally, exploring alternative triggers that induce platelet desialylation in ITP represents an interesting research direction, given substantial indication that other ITP plasma components and also platelet-directed CD8^+^ T cells may have platelet desialylating capacity, Figure 1. These unknown plasma components are likely immunologic factors, like complement or damage- or pathogen-associated patterns, that also activate platelets [102], although studies on the correlation between platelet activation and desialylation remain inconclusive [15,61,63]. Furthermore, the functional consequences of platelet desialylation warrant further examination, including the impact on coagulative function and intercellular interactions.

While in murine studies, clearance of desialylated platelets via the AMR in the liver is quite established, evidence for this hepatic route of platelet sequestration in patients with ITP is lacking. Clinical studies intertwining platelet desialylation rates, TPO levels, sequestration patterns, and platelet counts are necessary to further elucidate the contribution of the liver and spleen in both senescent and ITP platelet clearance. The current literature suggests that platelet desialylation may serve as a contributing factor for thrombocytopenia, rather than being a specific diagnostic or prognostic marker for ITP. While elevated platelet desialylation may reflect an active immune response in ITP and thus more severe disease, longitudinal studies are needed to understand the clinical significance of platelet desialylation in ITP. Lastly, studies on megakaryocyte desialylation in ITP have been limited and should be replicated in larger cohorts. Overall, this review highlights several promising research directions to further study the glycobiology in ITP, as outlined in Table.

**Table:** Immune thrombocytopenia glycobiology research agenda.

**Part 1: Platelet autoantibody glycosylation**
Development of new ITP platelet autoantibody isolation techniques to obtain a more enriched autoantibody fraction to be able to investigate platelet-specific IgG-Fc glycosylation.
Inclusion of context-factors in IgG glycosylation studies including age, sex, disease phase, total IgG glycosylation and lifestyle factors.
**Part 2: Platelet membrane glycosylation**
Investigation of platelet desialylation capacity of different ITP autoantibodies, also with the same antigen specificity.
Identification of factors in ITP sera without detectable antibodies leading to platelet desialylation.
Investigation of the desialylating capacity of platelet-specific CD8^+^ T cells in ITP.
Mechanistic studies focusing on the intracellular signaling pathways leading to platelet desialylation, stratified for different triggers.
Functional elucidation of platelet desialylation in relation to effects on immunosuppression and coagulation.
Clinical studies to the correlation between platelet desialylation, platelet sequestration site and thrombopoietin levels in patients with ITP.
Longitudinal assessment of platelet surface sialic acid before and after ITP treatments to correlate with treatment response, corrected for platelet counts.
**Part 3: Megakaryocyte membrane glycosylation**
The effects of (known) platelet desialylating ITP antibodies on megakaryocyte glycans and function.
Mapping of megakaryocyte desialylation in ITP compared to non-ITP thrombocytopenia and healthy controls.

Fc, fragment crystallizable; IgG, immunoglobulin G; ITP, immune thrombocytopenia.
